# Reassessing cardiometabolic health disparities among young adult sexual minority women: Evidence from a population-based cohort

**DOI:** 10.1371/journal.pone.0358185

**Published:** 2026-10-13

**Authors:** Kate Keenan, Stephanie D. Stepp, Alida Bouris, Alicia K. Matthews, Kristen Wroblewski

**Affiliations:** 1 Department of Psychiatry and Behavioral Neuroscience, University of Chicago, Chicago, Illinois, United States of America; 2 Department of Psychiatry, University of Pittsburgh, Pittsburgh, Pennsylvania, United States of America; 3 Crown Family School of Social Work, Policy, and Practice, University of Chicago, Chicago, Illinois, United States of America; 4 Department of Adult Health and Gerontological Nursing, Rush University, Chicago, Illinois, United States of America; 5 Department of Public Health Sciences, University of Chicago, Chicago, Illinois, United States of America; Christ College: CHRIST (Deemed to be University), INDIA

## Abstract

**Purpose:**

We compared objective measures of cardiometabolic health (CMH) between women who identify as lesbian/gay or bisexual (LGB) and women who identify as heterosexual. In addition, we aimed to test whether group differences in stress reactivity partially explained disparities in CMH health.

**Methods:**

Participants from a longitudinal study were identified for enrollment in 2017. The LGB group self-identified as LGB in 2017 (n = 239), and the heterosexual group identified as straight or heterosexual in each year that sexual identity was asked (study years 2010–2017) (n = 160). CMH was assessed via measurements and biomarkers, and stress reactivity via heart rate variability and cortisol levels during a social stress test. Participants also provided self-reports of stress and health. We tested group differences using sexual identity at Study Year 17, at the time of the visit, and as a proportion of years identifying as LGB.

**Results:**

Group differences were tested using t-tests for continuous measures, logistic regression for dichotomous measures, and repeated-measures ANOVA for change over time. There were no significant differences in measures of CMH or stress reactivity by sexual identity group. LGB participants had worse self-reported health and higher levels of stress compared to heterosexual participants.

**Conclusion:**

We discuss several frameworks for interpreting the present results of no differences in objective markers of health in the context of the existing literature, including developmental, socio-political, and methodological frameworks, and suggest directions for research to link the previous research with the results of the current study.

## Introduction

Results from several large surveys are consistent in providing evidence of health disparities for lesbian/gay or bisexual (LGB) women, specifically disparities in cardiometabolic health. For example, based on results from national longitudinal studies LGB women have higher body mass indices (BMI) and are at increased risk for diabetes compared to heterosexual women [1,2]. Findings from the Los Angeles County Health Survey (LACHS) of women showed that compared to heterosexual women, LGB women more often reported having a diagnosis of heart disease [3]. These data on middle-aged women suggest that important health disparities may exist in cardiometabolic health conditions for which the associated morbidity and mortality are high [4].

The impact of acute and chronic stress exposure on systems involved in maintaining health is well documented in correlational and experimental human and animal studies [5–8]. According to the Minority Stress Theory [9], members of minority groups are more likely than those from majority groups to experience stress, including discrimination stress, which likely impacts health systems [9,10]. For example, as early as adolescence, discrimination and unfair treatment because of minority status (e.g., race, poverty) are associated with health risks, including higher ambulatory blood pressure and C-reactive protein (CRP) [11,12].

These results are useful for generating hypotheses regarding mechanisms that may explain greater health risks among other minority populations, including LGB women. Self-report data support the hypothesis that experiences of discrimination are also associated with poor health among sexual minorities with diverse gender identities [13]. Meyer and colleagues (2008) showed that compared to heterosexual women and men, and to gay and bisexual men, LGB women report greater exposure to stressful life events and to discrimination-related life events [14]. Cochran and Mays (2007) used data from the CQLS to explore associations between psychological distress, sexual minority status, and self-reported physical health; no differences in the physical health of LGB and heterosexual women were observed after accounting for concurrent level of distress [15]. Hatzenbuehler and McLaughlin [16] reported that LGB young adults who were raised in high-stigma environments as adolescents evidenced a blunted cortisol response to a social stressor compared to those from low-stigma environments. Thus, the theoretical and empirical support for testing the mechanistic role of discrimination stress in the development of health disparities for sexual minority women is strong.

It is now evident via studies such as the Coronary Artery Risk Development in Young Adults (CARDIA) Study and other longitudinal studies of adolescents and young adults that individual differences in cardiovascular and metabolic health markers are observable in young adulthood and prospectively and reliably associated with later disease [17–20]. In fact, risk scores computed from modifiable factors measured at a single time point before age 20 (e.g., elevated cholesterol levels, hypertension) performed better than those computed 15 years later in predicting the extent of coronary atherosclerosis at age 35 [19]. Furthermore, the incidence of hypertension, pre-diabetes, and Type II diabetes among young adults is increasing in the population at large [20–22]. Thus, testing the hypothesis that LGB women evidence poorer cardiometabolic health than heterosexual women, and that such differences are observable in early adulthood, is critical from a prevention perspective.

Taken together, these data underscore the impact of stress on health and the critical need for risk reduction in early adulthood to prevent cardiac and metabolic diseases, especially for sexual minority women who appear to be at particularly high risk. In the present study, we aimed to expand the exisiting research on disparities in CMH for LGB women by including objective biomarkers of CMH, assessing stress regulation in response to a controlled laboratory stressor, and testing group differences using participants in a community-based sample of young adult women who have been participating in a longitudinal study that began when they were 5–8 years old. The inclusion of participants from the community avoided biases introduced by self-selection into studies and further provided the opportunity to use multiple waves of data to inform sexual identity. This study addressed two research questions. First, do young adult sexual minority women differ from heterosexual women on objective indices of cardiometabolic health? Second, is any such difference accounted for, in part, by group differences in stress reactivity? Our specific hypotheses are: 1: Compared to heterosexual women, LGB women will evidence greater health risks as indicated by objective measures of cardiometabolic health; and 2: The association between sexual minority status and health will be partially mediated by altered patterns of stress reactivity.

## Materials and methods

### Participants

The population-based sample of the Pittsburgh Girls Study (PGS) comprised 5- to 8-year-old females (by parent report) residing in the city of Pittsburgh in the year 2000 (study year 01). Participants were recruited via a stratified, random household sampling, with over-sampling of households in low-income neighborhoods [23]. Neighborhoods in which at least 25% of the families were living at or below the poverty level were fully enumerated, and a random selection of 50% of the households in all other neighborhoods were enumerated. The enumeration identified 3,118 separate households in which an age eligible female resided. From these households, families who moved out of state and families in which the girl would be age-ineligible by the start of the study were excluded. Of the 2,992 eligible families, 2,875 (96%) were successfully re-contacted to determine their willingness to participate in the study. Of those families, 85% agreed to participate, yielding a sample of 2,450, with a relatively equal distribution across the four age cohorts of 5-, 6-, 7-, and 8-year-olds. In-home interviews were conducted annually beginning in 2000.

The present study included a subsample of PGS participants who were identified for enrollment in 2017, which corresponded to study year 17, at which time participants were 21–24 years of age. We first determined the number of participants meeting inclusion criteria for the LGB group: currently self-identifying as lesbian, gay, or bisexual, living in Allegheny County, and having a Black, White, or multi-racial identity. Of the 317 participants meeting inclusion criteria, 239 (75%) enrolled, 57 (18%) were unable to be contacted or scheduled prior to the end of study enrollment, and 21 (7%) declined participation.

For the comparison sample, we randomly selected from participants who identified as heterosexual in each year that sexual identity was asked (study years 10–17), were living in Allegheny County, and who were frequency matched on age cohort and racial identity to the LGB group (two participants who requested to participate who were not in the random subsample were also included). Of the 228 eligible participants, 160 (70%) enrolled, 40 (18%) were unable to be contacted or scheduled prior to the end of study enrollment, and 28 (12%) declined to participate. This approach allowed us to generate an ecological valid heterosexual group that was distinct from the LGB group, within which some participants identified as heterosexual in some study years. There was no difference in age between those who did and didn’t enroll, but enrollment was higher for women who identified as Black compared to those who identified as White (77.6% versus 65.3%, *χ*^2^ [2] = 9.57, *p* = .002).

Of the 399 participants, 261 (65.4%) identified as Black, 126 (31.6%) as White, and 12 (3.0%) as Black and White. The original 5- and 6-year-old age cohorts each accounted for close to 30% of the participants; 23% and 18% were in the 7- and 8-year-old age cohorts, respectively. By design, the distribution of race and age cohort did not differ between the LGB and heterosexual groups.

Among the 239 participants who identified as LGB at the time of enrollment in 2017 (study year 17), 195 (81.6%) identified as LGB, 41 (17.2%) identified as heterosexual or straight, and 3 (1.3%) did not provide information on sexual identity at the time of the laboratory visit. Among the 160 participants who had reported a heterosexual identity in all annual interviews between study years 10–17, 144 (90%) identified as heterosexual or straight, 10 (6.3%) identified as LGB, and 6 (3.8%) did not provide information on sexual identity at the time of the laboratory visit.

We examined the variability in reporting across study years among the participants who identified as LGB in Year 17 by computing the proportion of interviews from years 10–17 in which an LGB identity was endorsed: the proportion ranged from 11% to 100%, with a median and mean close to 60%, and a mode of 100%. For participants who identified as LGB in Study Year 17 but not at enrollment (n = 44), the proportion ranged from 11% to 100%, with a median of 25%, a mean of 43% and a mode of 13%. Thus, in addition to testing differences in cardiometabolic health between groups based on study recruitment, we test identity at the time of the visit and identity as an ordinal measure of number of years of identifying as LGB and cardiometabolic health.

### Procedures

All participants completed the informed consent process, including providing written consent prior to data collection. The University of Pittsburgh Institutional Review Board approved all study procedures and consent documents. The study protocol included a laboratory visit during which participants completed questionnaires and participated in a controlled laboratory stress paradigm described in detail below. To control for circadian cortisol rhythms and optimize the ability to detect a response, laboratory visits occurred between 2:00 PM and 5:00 PM, when cortisol levels are typically low. Phlebotomists collected venous blood during a subsequent home visit, the morning following an overnight fast, for the assessment of CMH biomarkers. We assessed pregnancy via verbal report and urine test upon arrival to the lab, and re-scheduled laboratory visits and blood collection for at least 6 months following birth, or at least 9 weeks following pregnancy termination to avoid having to control for pregnancy in analytic models. Participants were reimbursed for their time at the rate of $100 for the lab visit and $100 for the blood draw, plus transportation costs. Data were collected between 09/28/2017 and 05/26/2022; 360 of the 399 participants (90%) completed the study visits prior to the COVID-19 shutdown.

### Measures

#### Sexual and racial identity.

A single question was administered to assess sexual identity: “Do you consider yourself to be heterosexual or straight, gay or lesbian, or bisexual.” A single question was used to assess racial identity with the option to select all applicable racial identities (n = 15).

#### Blood biomarkers.

Glucose was determined by electroimmunoassay (EIA) with a coefficient of variation (CoV) of 2.1%. Total cholesterol, high-density lipoprotein (HDL), and triglycerides were determined by conventional enzymatic methods (CoV, 1.3–2.1%). Insulin was measured by RIA procedure (CoV, 2.6%). CRP was determined turbidimetrically (CoV is 3.0%). All blood samples were assayed in the CLASS Laboratory at the University of Michigan.

#### Physical measurements.

Waist circumference was measured at the level of the umbilicus and hip circumference at the greatest expanse of the buttocks. Resting blood pressure, heart rate, and heart rate variability (the latter two averaged over a 5-minute period) were measured seated after 10 minutes of rest.

#### Stress reactivity.

The Trier Social Stress Test (TSST) was used to assess reactivity to a social stressor [24]. The TSST consists of a 5-minute preparation period, a 5-minute speech on discrimination, and a 2-minute mental arithmetic task. Prior to TSST administration, participants had a 20-minute baseline period to acclimate. During the 5-minute preparation, participants were instructed to prepare a speech describing a recent experience of discrimination [25]. The speech and serial subtraction tasks were delivered in front of a video camera and two judges. Following the stressor, a 60-minute recovery period was provided.

Saliva samples for cortisol assessment were collected at eight time points: upon arrival, at 10 minutes, 5 minutes, and 0 minutes pre-TSST, and at 20, 40, 50, and 60 minutes post-TSST. Saliva samples were collected using absorbent salivettes. Samples were stored in the laboratory at −40° C until assayed. On the day of testing, samples were thawed, centrifuged at 3,000 rpm for 10 minutes, and pipetted into test wells. All samples from each subject were assayed in the same batch to minimize variability, and with reagents from the same lot. Samples were assayed in duplicate using the Salimetrics HS Salivary Cortisol EIA Kit for unbound cortisol. This assay has a lower limit of sensitivity from.007 to 1.2 ug/dL. The average inter-assay variance is 3.9% and 7.1%, and the average intra-assay variance is 6.7% and 6.9% for high and low concentrations, respectively. The correlation between saliva and serum using the Salimetrics EIA Kit and the Coat-a-Count Serum Cortisol RIA kit is *r* = .96, *p* < .001.

Heart rate variability (HRV) was measured from a continuous time series of inter-beat intervals during 5 minutes of rest prior to the TSST, throughout the 7-minute task, and during 15 minutes of rest following the TSST. Prior to calculating HRV estimates, the digitized electrocardiogram signals were examined, and artifactual detections of R-wave occurrences were corrected. The square root of the mean of successive differences (RMSSD) in inter-beat intervals was used as a time-domain estimate of HRV.

Systolic and diastolic blood pressure were collected on the dominant arm using a standard, automatically inflating sphygmomanometer at eight time points: upon arrival, at 10 minutes, 5 minutes, and 0 minutes pre-TSST, and at 20, 40, 50, and 60 minutes post-TSST. Although blood pressure upon arrival was collected on all participants, blood pressure in response to the TSST was not included as part of the visit protocol until one year into data collection and thus is available for 70% of participants.

We tested whether cortisol, blood pressure, and heart rate differed at baseline as a function of alcohol, caffeine, nicotine, or prescription medication use in the past 12 hours, as reported by 144 (36%) participants. There was a trend toward a significant association between substance intake and baseline cortisol (*p* = .07); thus, substance use in the past 12 hours was included as a covariate in testing group differences in cortisol reactivity to the TSST.

#### Self-reported stress and health.

The 9-item Everyday Discrimination Scale (EDS) includes questions that begin with the following prompt: “In your day-to-day life how often have any of the following things happened to you?” followed by nine statements including: “You are treated with less courtesy than other people” and “People act as if they are afraid of you.” Items are scored on a 6-point Likert scale ranging from 1 (never) to 6 (almost every day). This version has demonstrated reliability and validity in young adults [26]; internal consistency for the EDS in the present study was strong (α = .89). Life Satisfaction was measured with the 5-item Satisfaction with Life Scale (SWLS) [27]. Items are scored on a five-point Likert scale (0 = never to 4 = very often). The SWLS has high internal consistency and temporal reliability: α = .89 in the present sample. The 10-item Connor-Davidson Resilience Scale (CD-RISC-10) asks respondents to what extent they agree with 10 statements about themselves (1 = never true to 5 = very often true) in order to assess their overall resilience (e.g., “I am not easily discouraged by failure”) using the past month as the reporting time frame [28]. Internal consistency as measured was high for the present sample (α = .90). The *Perceived Stress Scale* (PSS) measures individual perceptions about the nature of events and their relationship to the values and coping resources of an individual (e.g., “How often have you felt that you were on top of things?”) [29]. Each item is scored on a five-point scale from 0 (never) to 4 (very often), with positive items reverse-scored such that higher scores reflect higher levels of perceived stress. Internal consistency for the PSS in the present sample was strong (α = .80). The PROMIS Global Health measure assesses physical and emotional health [30]. The respondent reports their generic (as opposed to disease-specific) health on various scales (poor-excellent; not at all-completely; never-always; no pain-worst pain imaginable) for 10 items (α = .83).

#### Data analysis.

Blood biomarker and cortisol values were analyzed using log_10_ transformations; non-transformed values are used in Tables and Figures for ease of interpretation. We conducted *t* tests to determine which biomarkers and physical measurements, and which self-reported health and stress measures significantly differed between the LGB and heterosexual groups. We further tested associations between clinical indices of cardiometabolic risk using the following cutoffs: systolic blood pressure >120 mmHg, diastolic blood pressure >80 mmHg, total cholesterol >200 mg/dL, HDL-C < 50 mg/dl, triglycerides >150 mg/dL, glucose >99 mg/dL, and insulin >25 mIU/L. Logistic regressions were used to test differences in these clinical definitions of risk by sexual identity group. Repeated-measures analysis of variance (ANOVA) was used to test time and time-by-group effects on stress reactivity, as indicated by changes in systolic and diastolic blood pressure, heart rate variability, and cortisol output, assessed before and after the TSST; the cortisol model included substance used in the last 12 hours and time of day as covariates. A Greenhouse-Geisser correction was applied given that the dependence among the repeated measures. Analyses were conducted with SPSS Statistics version 31.0; the level of statistical significance was set at *p* < .05.

## Results

### Association of sexual identity with cardiometabolic biomarkers and physical measurements

There were no significant differences in blood biomarkers or physical measurements across sexual identity groups using identity data from study year 17, identity on the day of the lab visit, or the proportion of interview years in which the participant had identified as LGB. Data from study year 17 for the two sexual identity groups are presented in Table 1. There was no increase in the odds of a score falling in the clinical risk range as a function of sexual identity, using any of the three approaches to group assignment.

**Table 1:** Tests of differences in cardiometabolic biomarkers by sexual orientation subgroups.

	LGB	Heterosexual			
Blood biomarkers	Mean (SD)	Mean (SD)	Mean Diff (95% CI)	*t*	*p*
Triglycerides (mg/dL)	106.33 (80.58)	94.70 (45.00)	0.03 (−0.01–0.07)	1.29	.199
Cholesterol (mg/dL)	176.68 (30.68)	178.47 (35.40)	0.00 (−0.02–0.01)	−0.32	.750
HDL (mg/dL)	56.71 (12.75)	57.13 (13.62)	0.00 (−0.02–0.02)	−0.23	.766
Insulin (μIU/mL)	21.62 (18.70)	22.75 (22.25)	0.00 (−0.07–0.08)	0.10	.915
Glucose (mg/dL)	103.00 (32.02)	99.78 (20.82)	0.01 (−0.01–0.02)	1.05	.293
CRP (mg/mL)	3.40 (00.71)	3.40 (00.72)	0.00 (−0.14–0.14)	−0.02	.985
Physical measurements					
Heart Rate (BPM)	74.64 (10.10)	76.37 (10.53)	−1.72 (−3.88 – −0.42)	1.58	.115
HRV (RMSSD)	57.11 (36.60)	52.72 (33.30)	4.38 (−3.05–11.81)	1.16	.247
Systolic BP (mmHg)	110.49 (14.11)	109.58 (11.35)	0.91 (−1.74–3.55)	0.67	.501
Diastolic BP (mmHg)	76.76 (08.15)	71.65 (08.57)	0.86 (−0.57–2.80)	1.30	.194
Waist circumference (cm)	93.98 (21.19)	90.26 (18.37)	3.72 (−0.40–7.84)	1.78	.077
Hip Circumference (cm)	106.28 (20.94)	104.28 (18.57)	2.00 (−2.11–6.11)	0.96	.339

Note: HDL = high density lipoprotein; CRP = C-reactive Protein; RMMSD = root mean square of successive differences. Analyses of HRV and blood biomarkers were conducted with log_10_ transformed variables to address skewness.

### Association of sexual identity with stress reactivity

There was a significant effect of time on systolic (*F* [6.09, 1585.7] = 44.95, *p* < .001) and diastolic (*F* [5.26, 1336.05] = 46.52, *p* < .001) blood pressure, but no interaction of group x time (*p* values = .660 and.820 for systolic and diastolic blood pressure, respectively) (see Fig 1). There was a significant effect of time on heart rate variability (*F* [4.01, 1372.6] = 214.66, *p* < .001) and a trend for an effect of time on cortisol (*F* [2.52, 677.82] = 1.88, *p* = .070), but no interaction of group x time (*p* values = .139 and.450 for heart rate variability and cortisol, respectively) (see Fig 2). The pattern of results were similar using all three approaches to group assignment: identity data from study year 17, identity on the day of the lab visit, or proportion of interview years in which the participant had identified as LGB. Data using identity data from study year 17 are presented in Figs 1 and 2.

**Fig 1:**
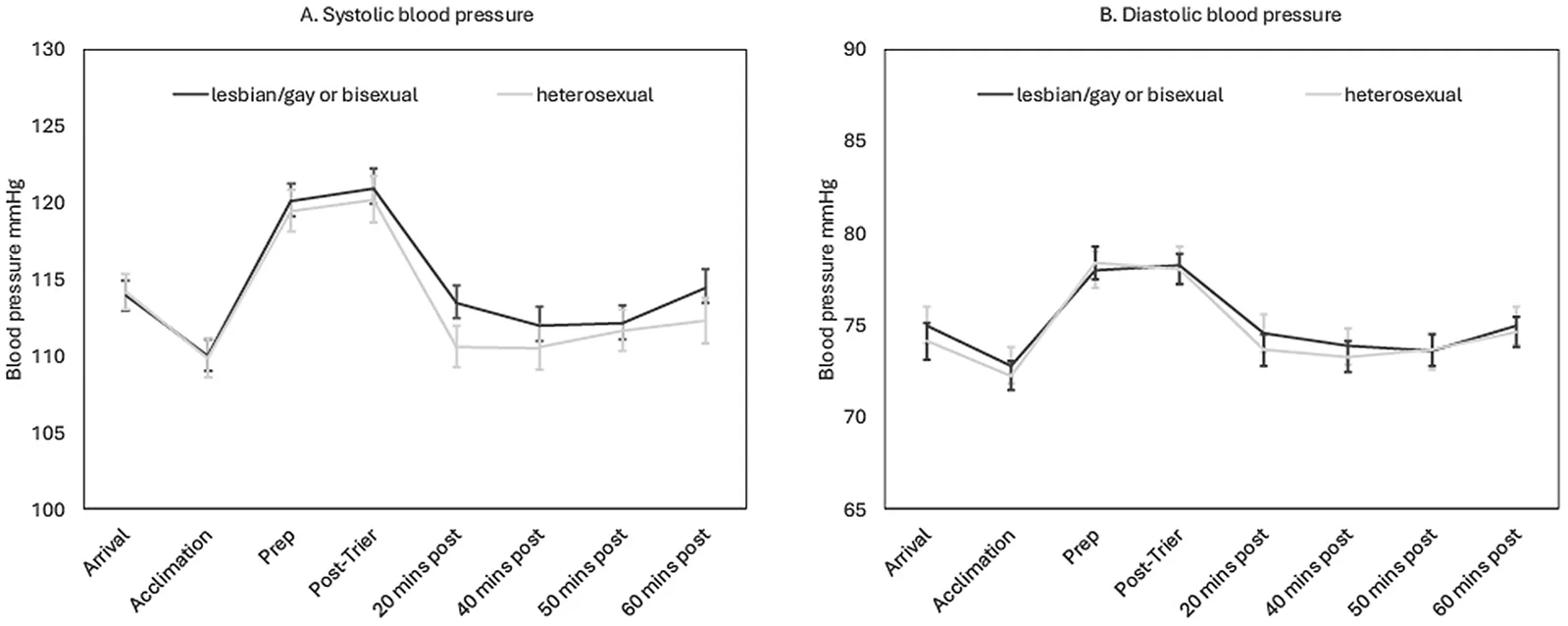
Average systolic (panel A) and diastolic (panel B) blood pressure at each interval relative to the Trier Social Stress Test. Separate lines are plotted for lesbian/gay or bisexual and heterosexual participants; error bars represent standard error of the mean.

**Fig 2:**
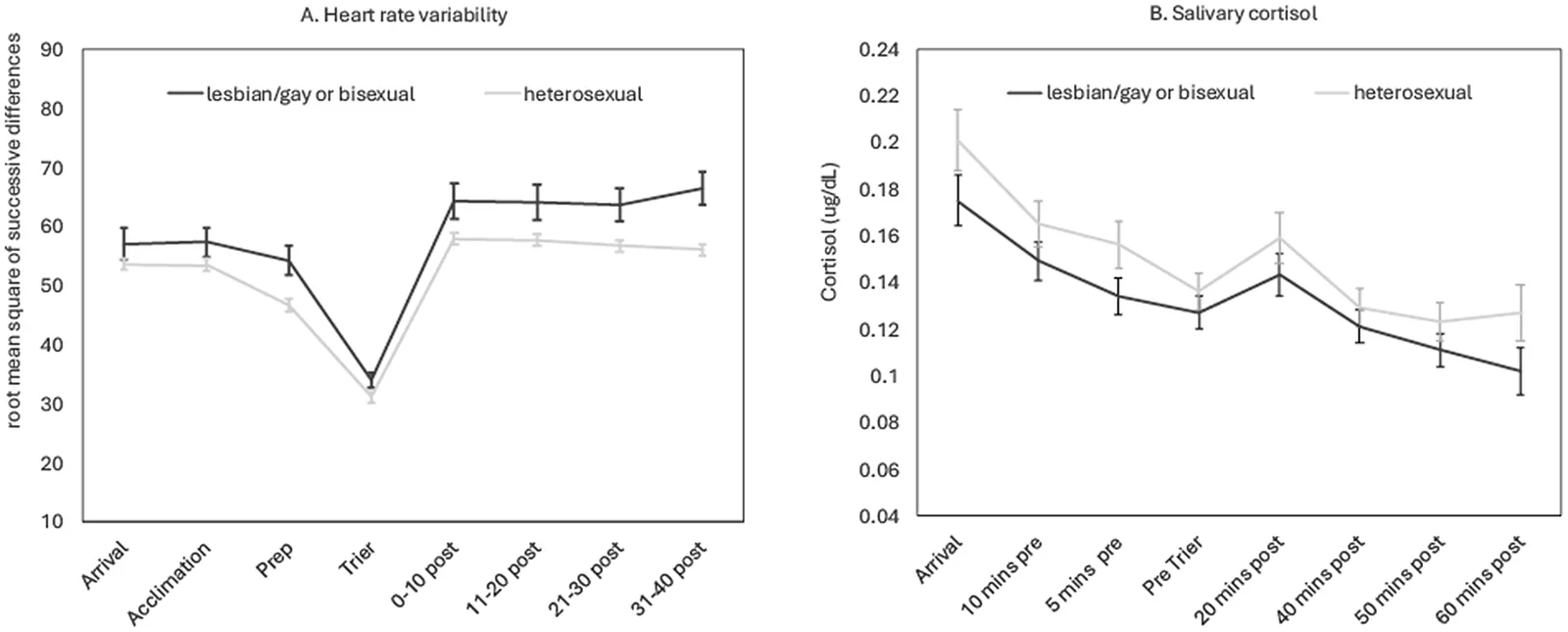
Average heart rate variability as indexed by root mean square of successive differences (panel A) and salivary cortisol levels (panel B) at each interval relative to the Trier Social Stress Test. Separate lines are plotted for lesbian/gay or bisexual and heterosexual participants; error bars represent standard error of the mean.

### Association of sexual identity with self-reported health and stress

All measures of self-reported health and stress were significantly different between participants who identified as LGB in Year 17 compared to those who identified as heterosexual in all study years except for resilience, for which no significant difference was observed (see Table 2); these effects were maintained in a multivariate model that included all five measures of health and stress.

**Table 2:** Tests of differences in self-reported health and stress by sexual orientation subgroups.

	LGB	Heterosexual			
	Mean (SD)	Mean (SD)	Mean Diff (95% CI)	*t*	*p*
Global Health	30.95 (6.14)	34.39 (5.90)	−3.44 (−4.65 – −2.23)	−5.58	<.001
Perceived Stress	20.24 (6.60)	17.58 (5.94)	2.65 (1.38–3.93)	4.09	<.001
Discrimination Stress	17.43 (5.78)	16.02 (5.46)	1.41 (0.27–2.54)	2.44	.015
Life Satisfaction	18.70 (7.28)	21.03 (7.09)	−2.33 (−3.78 – −0.89)	−3.17	.002
Resilience	37.00 (7.10)	37.91 (7.31)	−0.91 (−2.66–0.83)	−1.03	.306

Participants who identified as LGB reported poorer global health, higher perceived stress and discrimination stress, and lower life satisfaction. The global health measure also generates scores for physical and emotional health. As shown in Fig 3, the distribution of ratings of both physical (*χ*^2^ [4] = 15.00, *p* = .005) and emotional (*χ*^2^ [4] = 17.49, *p* = .002) health differed for LGB and heterosexual participants, with LGB participants endorsing less optimal health.

**Fig 3:**
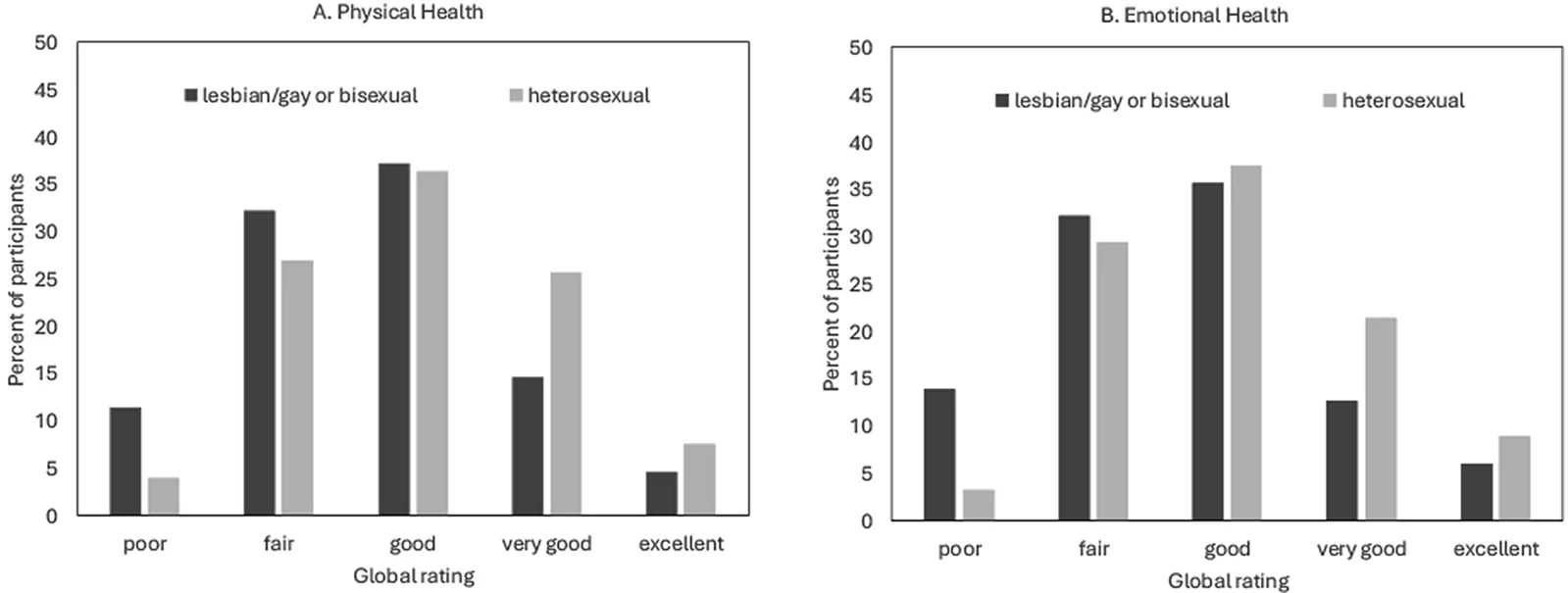
Percent of participants with self-reported physical (panel A) and emotional (panel B) health described as poor, fair, good, very good, or excellent. Separate bars presented for lesbian/gay or bisexual and heterosexual participants.

We further explored whether a Black racial identity moderates the associations between sexual identity and self-reported health and stress; no moderation effects were observed.

## Discussion

Contrary to our hypotheses, no significant differences were observed in biomarkers of cardiometabolic health or stress reactivity. Per self-report, LGB women reported lower overall health, less life satisfaction, a greater sense of uncontrollability of stressors, and higher levels of perceived discrimination. We consider several frameworks for interpreting the present results of no differences in objective markers of health in the context of the existing literature upon which our hypotheses were based. The first is from a developmental framework. The participants in the PGS study were on average 25 years of age and therefore younger than women in many of the previous studies. It is possible, therefore, that the lack of differences in objective health indicators in the current study is due to the developmental window within which health was measured. Moreover, the observed group differences in self-reported health and stress may serve as proximal signals of impacts on health systems that are not yet observable as compromised health. In other samples of young adults, experiences with stress were prospectively associated with later cardiometabolic health [19]. This hypothesis is worth interrogating to determine whether self-reports of health and stress can be used to identify LGB women who will later be at risk for compromised cardiometabolic health. Collecting follow-up data that includes the middle and later adult stages, when cardiometabolic health problems increase, would clarify whether the developmental timing of assessments played a significant role in these divergent findings.

The second is a sociocultural framework. Most policy changes for individuals identifying as sexual and/or gender minorities over the past 50 years have moved towards greater societal equity [31]. Marriage equality, anti-bullying programs, and gay-straight alliances (GSAs) in schools, along with expansions in national surveys that include LGBTQ identities, are cultural factors that may have had positive impacts on health. For example, following the Supreme Court’s Marriage Quality Act in 2015, disparities in health insurance as a function of sexual orientation decreased, such that by 2018 no disparities in coverage were observed [32]. In 2012, the U.S. Secretary of Education publicly endorsed GSAs in schools. In 2017, data were collected via the LGBTQ National Teen Survey, which included over 17,000 13–17-year-olds of diverse sexual and gender identities [33]. Using those data, Lessard and colleagues (2020) reported that compared to students in schools without GSAs, LGBTQ students at schools with GSAs reported lower levels of bullying, which in turn reduced the likelihood of adverse health outcomes such as sleep and eating problems [34]. Most studies showing significant health risks for LGB women were conducted in the early 2000s with participants who were, on average, 30 years and older. Individuals in the current sample were coming out 10–20 years later. Thus, the contrasting sets of findings may be attributable to better CMH health for LGB women via increased rights and protections. Our result on self-reported resilience provides some support for this interpretation. Despite higher levels of self-reported stress exposure and global health, LGB participants reported similar levels of resilience to those reported by heterosexual women. Equal levels of resilience, in part perhaps to changes in the social contract, may help to protect LGB women from compromised cardiometabolic health in early adulthood. If increases in health disparities follow a return of policies and programs that decrease equity for LGBTQ individuals, that would support a role of the socio-political environment on our divergent findings.

The third framework is a methodological one. Several of the earlier studies used self-report or medical record data and in some, sample selection was not representative. It is possible that, had there been opportunities to collect objective health data from representative samples, the results would also have shown no differences in cardiometabolic health. Although data suggest that adults provide reliable self-reports on some health problems, such as breast cancer [35], the overlap of self-reports and objective measures of cardiometabolic health is less robust. For example, using data from NHANES (average age = 45 years), the correspondence between objective measures and self-report of a diagnosis of hypertension, elevated cholesterol, or kidney disease was poor [36]. Similarly, correspondence between biomarker and self-report data collected from adults (average age = 46) recruited at a Minnesota State Fair was low, especially for high-density lipoprotein and fasting blood glucose levels [37]. Challenges in identifying representative samples also likely impacts generalizability and rigor of data. A review of the literature on sexual health research on lesbian and gay people revealed that over 80% of studies used convenience sampling, and that differences in age, education, sexual risk-taking, uptake of screening and testing, and self-reported psychological distress were observed between convenience and national probability samples [38,39]. All three of these frameworks likely lend some explanation for the disparate results between earlier studies and ours. Determining which of the three accounts for most of the variance will help shape future efforts to support the health of LGB women.

### Limitations

In addition, several limitations of the current study should be addressed in future research. The first is the limited selection of CMH biomarkers collected at a single time point. In an effort to cover several CMH domains, including systemic inflammation, blood sugar regulation, and dyslipidemia, our measurements lacked depth within each domain. Repeated measurement also would have increased the rigor of the CMH construct by reducing the effects of diet, sleep, and/or inflammation in response to infection on measured biomarkers. Second, we were unable to probe subgroup and intersectional identities on health due to the relatively small sample size. Although we explored whether Black and White race moderated the association between sexual identity group and self-reported health and stress, we did not have sufficient power to model the interactive effects of race and sexual identity fully, nor did we have adequate representation to compare health among lesbians, bisexual, and heterosexual women. In other studies, bisexual identity or behavior confers risk for poor cardiometabolic health [40,41]. We also do not have data on gender identity. PGS participants were enrolled based on parental report of female sex in childhood, but without self-report we could not determine whether current gender identity differs from the sex recorded at enrollment. Results from a meta-analysis of studies conducted in the US and the United Kingdom suggest that transgender individuals are at high risk for cardiometabolic health problems [42]. Finally, we observed group differences in self-reported health, but we did not assess specific health domains. Problems with sleep, diet and appetite, and energy and activity levels are critical elements of health that are likely early disruptors of cardiometabolic health.

In summary, results showing a lack of association between sexual identity and objective measures of cardiometabolic health and stress reactivity, but group differences in self-reported health and stress are intriguing and, in the context of the existing literature, can help guide the next set of hypothesis testing regarding the impact of sexual identity on women’s health.

Several future research priorities follow from these findings. First, cardiometabolic risk typically becomes detectable in the fourth decade of life; extended follow-up of this and comparable cohorts is needed to determine whether the null findings observed here reflect a genuine absence of disparity or a disparity that has not yet emerged. Second, expanded assessments of stress reactivity that include ambulatory measures of stress physiology along with ecological momentary assessment or daily-diary logs may reveal differences in patterns of reactivity to specific stimuli. Third, cohort studies that have sufficient representation to model the effects of intersecting identities across sexual, racial and gender identity are needed. Fourth, measures of protective factors, such as community connectedness and identity affirmation, may help in interpreting differences in the association between sexual identity and subjective versus objective health indices.
